# Novel Extraction Method for Combined Lipid and Metal Speciation From *Caenorhabditis elegans* With Focus on Iron Redox Status and Lipid Profiling

**DOI:** 10.3389/fchem.2021.788094

**Published:** 2021-12-09

**Authors:** Bastian Blume, Michael Witting, Philippe Schmitt-Kopplin, Bernhard Michalke

**Affiliations:** ^1^ Research Unit Analytical BioGeoChemistry, Helmholtz Center Munich - German Research Center for Environmental Health (GmbH), Neuherberg, Germany; ^2^ Metabolomics and Proteomics, Helmholtz Center Munich, Neuherberg, Germany; ^3^ Chair of Analytical Food Chemistry, TUM School of Life Science, Technical University of Munich, Freising, Germany

**Keywords:** iron (II), iron (III), extraction, lipidomics, metal speciation, *C. elegans* model

## Abstract

Parkinson´s disease progression is linked to iron redox status homeostasis via reactive oxygen species (ROS) formation, and lipids are the primary targets of ROS. The determination of iron redox status *in vivo* is challenging and requires specific extraction methods, which are so far tedious and very time-consuming. We demonstrated a novel, faster, and less laborious extraction method using the chelator ethylene glycol l-bis(β-aminoethyl ether)-N,N,N′,N′-tetra acetic acid (EGTA) as a stabilizing agent and synthetic quartz beads for homogenization under an argon atmosphere. Additionally, we combined the metal extraction with a well-established lipid extraction protocol using methyl-tert-butyl ether (MTBE) to avoid the problems of lipid precipitation in frozen samples and to determine lipid profiles and metal species from the same batch. The nonextractable matrix, such as the debris, is removed by centrifugation and digested to determine the total metal content of the sample as well. Lipid profiling using RP-LC–MS demonstrated high accordance of the modified extraction method to the reference method, and the organic solvent does not affect the iron redox status equilibrium. Furthermore, rigorous testing demonstrated the stability of the iron redox status equilibrium during the extraction process, secured by complexation, inert atmosphere, fast preparation, and immediately deep frozen extracts.

## Introduction

Parkinson´s disease is the second most common neurodegenerative disorder, which affects about 1 percent of the population above 65 years. Clinical symptoms occur after about 60 percent of dopaminergic neurons in the substantia nigra are lost (Adams Jr, Chang et al., 2001). Risk factors are commonly connected to genetic background, pesticides and heavy-metal exposure, drug intake, viral infection, or physical damage. Mostly, a combination of different risk factors leads finally to the onset of disease (Adams Jr, Chang et al., 2001; Klein and Westenberger 2012). The underlying mechanisms are not fully understood, but an imbalance in forming and scavenging of reactive oxygen species (ROS) is accepted to play a crucial role in Parkinson´s disease progression (Kolodkin, Sharma et al., 2020). ROS are formed enzymatically by the electron transport chain reaction in the inner mitochondrial membrane or *via* cytochrome P450 by one-electron reduction of O_2_ (Quinlan, Perevoshchikova et al., 2013). The formed superoxide anion (O_2_^**-**^) is dismutated spontaneously or catalyzed by superoxide dismutase (SOD) to H_2_O_2_ (Wang, Branicky et al., 2018). Ferrous iron [Fe (II)] has a crucial role in the formation of the hydroxyl anion (OH^−^), and the hydroxyl radical (OH^**∙**^) forms H_2_O_2_ via the Fenton reaction. The OH^**∙**^ is highly reactive and mainly responsible for oxidative damage in lipid membranes (Graf, Mahoney et al., 1984; Gaschler and Stockwell 2017). Disruption in the iron redox state results in formation of Fe (II) in elevated concentration, thus accelerating the formation of the hydroxyl radical. Therefore, the iron redox state and the possibly caused lipid peroxidation in biological systems are of high interest for enlightening cellular mechanisms in Parkinson´s disease progression. The nematode *Caenorhabditis elegans* (*C. elegans*) has become a widely used model organism to probe for underlying mechanisms in neurodegenerative disorder progression, since basic cellular mechanisms are well-conserved in the nematode with 60–80% of homologous genes (Kaletta and Hengartner 2006; Leung, Williams et al., 2008). The nervous system of the nematode is relatively simple with around 302 neurons including dopamine (DA), acetylcholine (ACh), gamma-aminobutyric acid (GABA), serotonin (5-HT), glutamate, and others (White, Southgate et al., 1986). Short reproduction cycles of only 3 days allow to generate sufficient biomass for several treatment experiments in a short period. Hence, *C. elegans* is the perfect organism to establish new analytical methods and enable the enlightenment of basic mechanisms in neurodegenerative disorders (Chakraborty, Bornhorst et al., 2013). However, biological variability regarding iron redox and lipid metabolisms lead to relatively high batch to batch variances, which worsens the significance of the results. A usual but tedious solution to overcome this problem is performing many replicates, sample pooling, and extended comparison with control groups. So far, for each analytical method, separate sample preparation protocols are required increasing the number of samples as well as the amount of time. The extraction and speciation of the iron redox state from biological systems, such as *C. elegans*, is challenging because of low iron concentration and the sensitivity of Fe (II) to environmental oxygen during extraction. Nevertheless, the extraction method developed by J. Diederich and B. Michalke (Diederich and Michalke 2011) achieves the extraction of native iron and manganese species from the brain and liver tissue by working under the argon atmosphere using a *Potter-Elvehjem* tissue homogenizer. While the method secures the stability of iron species during extraction, the preparation is time-consuming and the throughput is low, since only one sample can be prepared at the same time. The speciation is carried out by capillary electrophoresis–induced coupled mass spectrometry (CE–ICP-MS) using 20 mM HCl as an electrolyte to prevent iron precipitation or adsorption on the capillary wall (Michalke, Willkommen et al., 2019) as well as by high-pressure liquid chromatography–ICP-MS (HPLC–ICP-MS) (Solovyev, Vinceti et al., 2017). A widely used and accepted extraction method was developed by Matyash et al. (Matyash, Liebisch et al., 2008), which used methyl-tert-butyl ether (MTBE) and methanol as extraction media instead of carcinogenic halogenated solvents. Yields and recoveries were similar to the lipid extraction protocols by Bligh and Dyer (Bligh and Dyer 1959) and Folch (Folch, Lees et al., 1957). Consequently, use of this extraction protocol over the last decade has increased.

Lipid profiling is carried out with liquid chromatography–MS (LC–MS) with the normal or reversed-phase. The identification of lipids is usually achieved by MS/MS spectra and matching with databases (Hermansson, Uphoff et al., 2005; Sommer, Herscovitz et al., 2006; Yetukuri, Katajamaa et al., 2007). Until now, no combined extraction protocol for ion redox speciation and lipid analysis has been published. However, there is an eminent need since both are strongly interlinked. Here, we report a combined extraction protocol, which allows simultaneous extraction of metal species. We particularly focused on iron redox state and lipid extraction in increased throughput. This extraction protocol is currently the missing tool for monitoring the lipidome of the same nematode population, specifically the oxidized lipids and iron redox state homeostasis. Previously mentioned limitations such as batch to batch variations and the amount of time spent on extraction are highly reduced. Additionally, the problematic of precipitating lipids and proteins in the metal containing aqueous phase after freezing and thawing, by separating lipids and metal species forehand will be avoided.

## Material and Methods

### Materials

Ethylene glycol l-bis(β-aminoethyl ether)-N,N,N′,N′-tetra acetic acid (EGTA) tetra sodium salt was purchased from Santa Cruz Biotechnology, Inc. (Dallas, United States). 3-morpholinopropane-1-sulfonic acid (MOPS) from MP Biomedicals (Illkirch, France) and sodium chloride (NaCl) of analytical grade from Fluka^®^ Analytical (Munich, Germany) were purchased. Agar granulate was ordered from BD Diagnostics (Franklin Lakes, United States). MTBE, acetonitrile (ACN), formic acid (Fa), and methanol (MeOH) of LC–MS grade were purchased from Sigma-Aldrich (Darmstadt, Germany). Butylhydroxytoluol (BHT) and cetyltrimethylammonium bromide (CTAB) were ordered from Th. Geyer GmbH and Co. KG (Renningen, Germany). Synthetic quartz beads (0.315–0.5 mm) were purchased from Gaßner Glastechnik GmbH (Oberhaching, Germany). HNO_3_ was purchased from Carl Roth GmbH und Co. KG (Karlsruhe, Germany) and purified by sub-boiling distillation. The metal extraction buffer (MEB) was prepared by dissolving exactly weighted respective amounts of MOPS, NaCl, and EGTA in MiliQ^®^ water to achieve the final concentration of 5, 6, and 1 mM, respectively. Fe (II) and Fe (III) standard solutions were prepared by dissolving 1 g/L FeCl_2_ in 100 mM HCl and FeCl_3_ in 1 mM HCl, respectively. Stock solution was aliquoted, kept under argon at −20°C, and were thawed and diluted shortly prior to usage. The dilution was carried out in the MEB.

### *C. elegans* Culture

The wild-type N2 strain of *C. elegans* was maintained and handled at 20°C according to previously published protocol (Brenner 1974). *Escherichia coli OP50 (E. coli),* cultivated in the Luria–Bertani medium (LB-medium), was used as a food source. Worms were bleached for age synchronization with basic hypochlorite solution as described previously by WB (WB 1988). Hatched L1 was fed with *E. coli* for 2 hours, washed two times with the 10 ml M9 buffer (22 mM KH_2_PO_4_, 22 mM Na_2_HPO_4_, 85 mM NaCl, and 1 mM MgSO_4_), incubated for 20 min in M9, and washed three times with 10 ml Milli-Q^®^ water. An amount of 100 k L1 was aliquoted, the liquid was aspirated, and the samples were frozen in liquid nitrogen and stored at -80°C until extraction.

### Extraction

#### Extraction According to Matyash et Al

For the lipid extraction, 50 µl MeOH was added to a pellet corresponding to 100 k L1 larvae and mixed. The suspension was transferred to 2-ml beading tubes filled with 200 mg quartz beads. This step was repeated once to transfer the pellet quantitatively. Then, 300 µl MTBE was added, the lids were closed, and the samples were incubated at 25°C for 1 h. By adding 100 µl water and incubating for 10 min at room temperature (RT), the phase separation was induced. The upper organic phase was collected after centrifugation at 10.000 *g* for 15 min, and the lower phase was reextracted with 200 µl MTBE/MeOH/H_2_O (10:3:2.5, v/v/v). Additionally, 50 µl BHT solution (58.5 mM in MeOH) was added to the combined organic phases. The combined organic phase was dried in a vacuum centrifuge for 30 min. The pellet was dissolved in isopropanol/acetonitrile/water (6:3.5:0.5, v/v/v) for LC–MS measurement.

#### Modified Extraction for Metal Species and Lipids

All buffers and solvents were prepared freshly and stored in the dark at 4°C until use. Before extraction, all buffers and solvents were degassed, and additionally, all buffers, solutions, and vials were flushed with argon for 1 h. Extraction was carried out by adding 100 µl MEB/Methanol (2:1, v/v) and 50 µl BHT solution (58.5 mM in MeOH) to a pellet corresponding to 100 k L1 larvae and mixed. The suspension was transferred to 2-ml beading tubes filled with 200 mg quartz beads. This step was repeated once to transfer the pellet quantitatively. Then, 300 µl MTBE was added; the samples were overlaid with Ar and stored on ice, lids were closed, and the sample was homogenized at −10°C at 6,000 rpm with 3 × 20 and 30 s pause, using a Precellys bead tube homogenizer, equipped with a cryo unit. Phase separation was induced by adding 100 µl MEB and incubated for 5 min at RT and centrifuged at 10.000 g for 10 min. The upper organic phase was collected, and the lower phase was reextracted with 200 µL MTBE/MeOH/MEB (10:3:2.5, v/v/v). The aqueous phases were carefully aspirated and immediately frozen in liquid nitrogen, whereas 300 µl MEB was added to the pellet. After mixing and centrifugation at 10.000 *g* for 5 min, the aqueous phase was aspirated, combined with the previous one, and frozen in liquid nitrogen. The frozen samples were overlaid with Ar and stored in liquid nitrogen. The combined organic phase was dried in a vacuum centrifuge for 30 min. The pellet was dissolved in isopropanol/acetonitrile/water (6:3.5:0.5, v/v/v) for LC–MS measurement.

### Metal Quantification in Aqueous and Organic Worm Extracts and the Pellet

400 µl aqueous and organic phases were diluted with 2% HNO_3_ up to 4 ml. The worm pellet and the quartz beads were transferred to 10-ml microwave digestion tubes using 1 ml mixture of subboiled HNO_3_, 30% H_2_O_2_, and water (1:1:2, v/v/v) and digested in total for 20 min (5 min heat up, 10 min at 200°C and 27 bar, and 5 min cooling down). The digested samples were diluted up to 10 ml with 2% subboiled HNO_3,_ mixed with three pipette strokes, and 5 ml was aspirated for measurement, after quartz beads have settled down. The quantification was carried out with inductively coupled plasma atomic emissions spectroscopy (Optima 7300 DV, PerkinElmer, Toronto, Canada) for the elements Fe, Al, Cu, Mn, and Zn (Table 1). Control (QC) and blank measurements were performed periodically after 10 measurements as well as before and after measuring the samples with the high organic content.

**TABLE 1:** Lines used for ICP-AES determination of selected elements.

Chemical	Emission line λ in nm
Al	167.022; 396.153
Cu	324.752
Fe	259.939
Mn	257.610
Zn	313.857

### Lipid Profiling by RP-LC–MS/MS

Lipid profiling was performed on a Sciex ExionLC AD system coupled to a Sciex X500R QToF-MS (MDS Sciex, Concord, Canada) equipped with an electron spray ionization source (ESI) operated in the negative and positive mode. Lipid separation was achieved on a Waters CORTECS UPLC C18 column (150 mm × 2.1 mm ID, 1.6 µm particle size) using a linear gradient eluant A (40% H_2_O, 60% ACN, 0.1% NH_4_FA) to eluant B (90% isopropanol, 10% ACN, 0.1%, NH_4_Fa) with a flow rate of 0.25 ml/min and column temperature of 40°C (Witting, Maier et al., 2014).

The MS was calibrated daily and every six samples to maintain high mass accuracy and operated in information dependent acquisition (IDA) using an ionization voltage of -4.5 and 3.8 kV. The m/z range was set from 50 to 1,500 Da. MS/MS information was acquired with IDA selecting 10 precursors and a collision energy of −35 and 35 eV with a collision energy spread of 15 eV.

### Iron Redox State Speciation

The aqueous phase was thawed, mixed, transferred to 300-µl sample vials, and capped. The experimental setup, including the interface between capillary electrophoresis (CE-700, prince technologies, Emmen, Netherlands) and ICP-MS (NexION 300 D, PerkinElmer, Toronto, Canada), was arranged as previously described (Michalke, Willkommen et al., 2019). An uncoated fused silica capillary with the dimensions of 100 cm × 50 µm inner diameter (ID) was used. The electrolyte was 50 mM MOPS (pH 6.2) with 0.5 mM CTAB. The capillary voltage was set to -15 kV, with an external pressure of 700 mbar to induce an additional flow. Before each run, the capillary was flushed at 4 bar with 10% HCl for 1 min, 0.5 M NaOH for 1 min, MiliQ water for 2 min, and the background electrolyte (BGE) for 5 min. Ammonium acetate in the concentration of 20 mM was used as a sheath liquid (Table 2).

**TABLE 2:** Conditions for capillary electrophoresis and Fe (II)/Fe (III) Speciation.

Chemical	Condition
10% HCl	2 bar, 1 min
0.5 M NaOH	2 bar, 1 min
H_2_O	2 bar, 2 min
BGE	2 bar, 5 min
Sample	150 mbar, 5 s
BGE	700 mbar, 5 min, -15 kV

The ICP-MS was used in the dynamic reaction cell (DRC) mode with ammonia as a reaction gas. The isotopes ^56^Fe and ^57^Fe were measured. The flow parameters of lenses, collision cell, carrier gas, plasma gas, and auxiliary gas were tuned for maximum sensitivity, low oxide ratio of <1.0% (^140^CE^16^O/^140^CE^+^), and double-charged ratio <1.5% (^140^Ce^++^/^140^Ce^+^) with background counts of <0.1 cps. The RF power was set to 1250 W, the plasma gas was set to 16 L Ar/min, the nebulizer gas was set to 0.94 L Ar/min, the dwell time was set to 50 ms, and the ammonia gas flow was set to 0.58 ml NH_3_/min. Operating software for MS was Syngistix from PerkinElmer (Toronto, Canada) and for CE was DAx-3D from prince (Emmen, Netherlands).

### Data Processing and Lipid Annotation

RP-LC–MS data were reprocessed using Genedata Expressionist for MS 15.5 (Genedata AG, Basel, Switzerland), which included chemical noise subtraction, peak detection, isotope and adduct clustering, and MS/MS peak detection. MS1 data were exported to .xlsx for further processing. MS2 data were exported as .mgf files and further processed in R using an in-house annotation workflow (https://github.com/michaelwitting/MetaboAnnotationGenedata). Lipids were putatively annotated by matching m/z values against an in-house *C. elegans* database and LipidMaps CompDB (Fahy, Subramaniam et al., 2009) as well as MS2 spectra data against LipidBlast (Kind, Liu et al., 2013) and an in-house *C. elegans* lipid database. Annotations were manually verified, and only verified annotations were reported. Any further data processing and generating graphs were carried out with the open source tool RStudio (Version February 1, 1335, RStudio, Inc.); and packages: ggplot2, ggpubr, openxlsx, reshape2, gridExtra, scales, grid, plyr, dplyr and tidyverse, mcr, and outliers.

### Statistical Analysis

For comparing two methods, we used the statistical test according to Pearson with *p* values below 0.05 considered to be statistically significant (Benesty, Chen et al., 2009). The Passing and Bablok regression was performed to compare the intensities of features for both methods with a confidence interval (CI) of 95% for the intercept and the slope (Passing and Bablok 1983). The error bars in bar plots present the standard error of the mean (SEM) obtained from three measurements. The confidence area was calculated according to the following equation (Eq. 1) with the number of samples (*n)*, the student factor for *n* variables and confidence probability of 0.95 (*t)*, and the standard deviation (*s)*:

$$\mu_{u/d}=\;\bar{x}\pm t\;\frac{s}{\sqrt{n}}$$ (1)

For the lipid profiling, only annotations identified in all triplicate and with a coefficient of variation (CV) <30% were accepted as valid features and were used for further statistics. The intensities of features in one lipid class were tested for normal distribution using the Shapiro–Wilk test. In case of normal distribution, the features of both extraction methods were compared using the independent two-tailed Student´s *t*-test (Student 1908).

## Results and Discussion

### Lipid Profiling

The lipidome of *C. elegans* includes a wide range of different lipid classes and sub classes such as fatty acids and amides, glycerophospholipids, sphingolipids, glycerolipids, steroids and related substances, glycolipids, prenol lipids, and additionally small classes (Witting and Schmitt-Kopplin 2016). The glycerophospholipids are the main components of biological membranes and therefore of high interest for determining potential cell damages caused by reactive oxygen species (ROS), whose alteration is typical for Parkinson’s disease progression (Kolodkin, Sharma et al., 2020). For the determination of changes in the lipid profile of the *C. elegans*, it is mandatory that the combined extraction method extracts the main lipid classes as good as the reference method (Matyash, Liebisch et al., 2008). Therefore, we compared the total number of annotated lipids and their intensities of selected lipid classes, with predominant focus on the glycerophospholipids: phosphatidylcholine (PC), lysophosphatidylcholine (LPC), phosphatidylethanolamine (PE), ether-linked PCs (PC-O) and PEs (PE-O), lysophosphatidylethanolamine (LPE), phosphatidylinositol (PI), phosphatidylglycerol (PG), phosphatidylserine (PS), diglyceride (DG), triacylglyceride (TAG), and other lipid classes.

In total, 849 lipid features were detected. A total of 822 in Matyash and 845 in the combined extraction protocol were detected. From these, 818 were found in both. The number of annotations based on MS2 data in the positive mode was 525 for the modified version and 505 for the reference method. The predominant species are the TAGs with 243 and 231 annotations, followed by the PCs with 125 and 123 annotations, and 63 and 47 annotations for the PEs. PC-Os, PEOs, LPEs, and other annotations are <16 for each of the classes (Table 3). The differences in the number of annotated lipids are considered as marginal, and the modified protocol has more annotations in total as well as more or the same annotations in the sub groups.

**TABLE 3:** Number of annotations in the positive mode for lysophosphatidylcholine (LPC), phosphatidylcholine (PC), ether-linked PCs (PC-O), phosphatidylethanolamine (PE), ether-linked PEs (PE-O), phosphatidylinositol (PI), diglyceride (DG), triacylglyceride (TAG), and other lipid classes.

Lipid class	LPC	PC	PC-O	PE	PE-O	PI	DG	TAG	Other lipid class
**Modified extraction**	9	125	5	47	16	5	21	243	54
**Matyash et al**	9	123	4	47	16	2	21	231	52

In the negative mode, the total annotations for the combined extraction method were 320 and for the reference method were 317. The predominant species are PEs with 106 and PCs with 94 annotations. Three PSs, which were annotated for the combined extraction method, could not be annotated for the reference extraction because of intensities lower than the threshold of 200 (Table 4). Conclusively, the comparison of the number of annotations, in the positive and negative ionization mode, shows no significance difference between the reference and the modified protocol. The marginal changes in the positive mode in the number of annotations might be explained by differences in the intensity, which lead to undergoing the threshold and conclusively no annotation. Therefore, we compared the absolute intensity for the annotated lipids for both extraction methods.

**TABLE 4:** Number of annotations in the negative mode for lysophosphatidylcholine (LPC), phosphatidylcholine (PC), ether-linked PCs (PC-O), phosphatidylethanolamine (PE), ether-linked PEs (PE-O), lysophosphatidylethanolamine (LPE), phosphatidylinositol (PI), phosphatidylserine (PS), phosphatidylglycerol (PG) and other lipid classes.

Lipid class	LPC	PC	PC-O	PE	PE-O	LPE	PI	PS	PG	Other lipid class
**Modified Extraction**	4	94	4	106	68	13	4	3	8	16
**Matyash et al**	4	94	4	106	68	13	4	0	8	16

**FIGURE 1:**
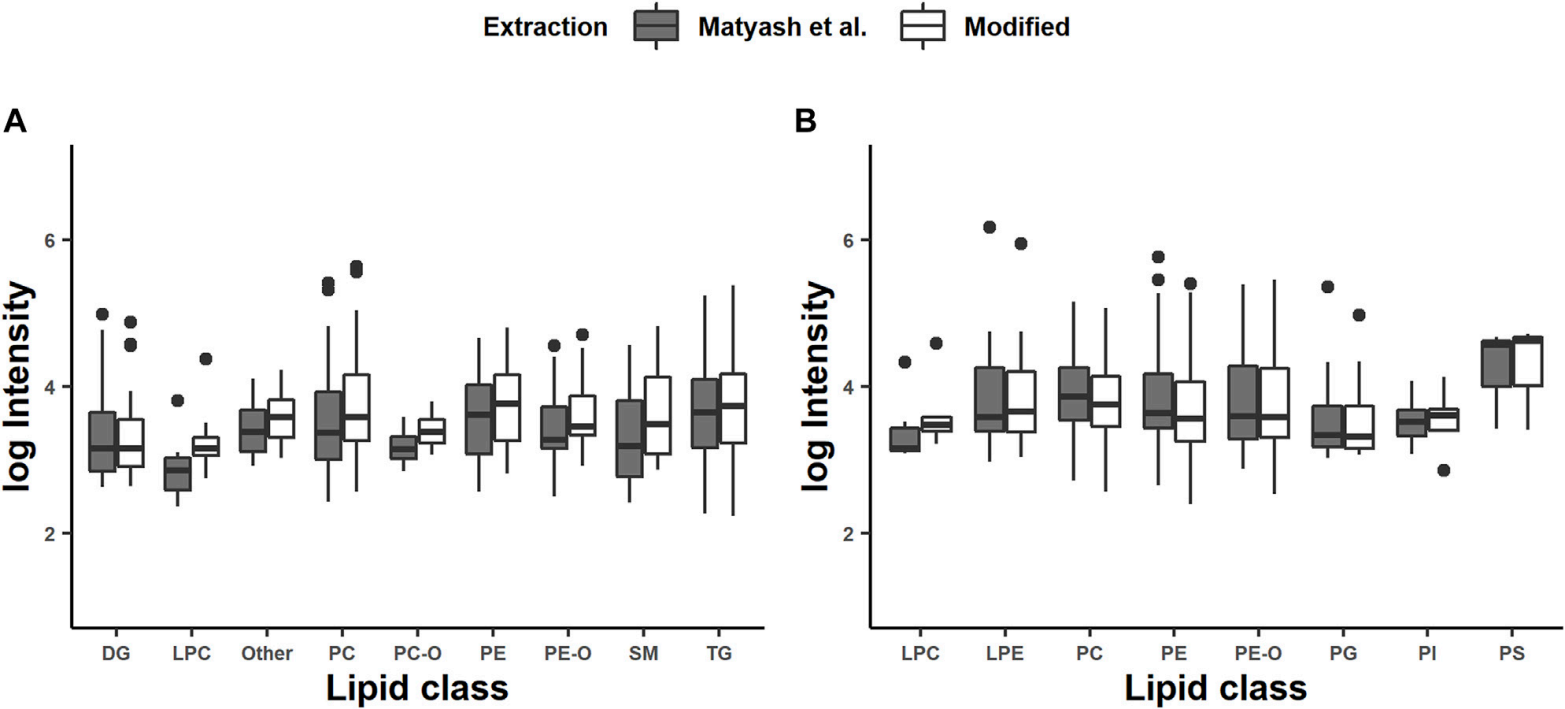
Intensities of chosen lipid classes generated in positive **(A)** and negative **(B)** modes: diglyceride (DG), lysophosphatidylethanolamine (LPE), phosphatidylcholine (PC), phosphatidylethanolamine (PE), phosphatidylinositol (PI), phosphatidylglycerol (PG), phosphatidylserine (PS), sphingomyelin (SM), triacylglyceride (TAG), and other lipid classes. The intensities of identified lipids are represented as averages from triplicate determination.

Nonvolatile or less volatile compounds, such as salts, ion-pairing agents, endogenous compounds, drugs, and metabolites, change the electron spray droplet properties, which results in ion suppression (Thomas 2003). Therefore, we studied the effect of the MEB and the shortened incubation time on the intensity of the features. The range of intensities was from 200 to 1 × 10^7^ counts, which are multiple orders of magnitude. Consequently, we applied the logarithmic intensities against the lipid class (Figure 1). Interestingly, the average intensities for all lipid classes were higher in the modified extraction method for the positive mode and without noticeable differences in the negative mode. We further applied Student´s *t*-test for the intensities and the coefficient of variation within one lipid class. The differences between the intensities within the lipid classes were not significant except for the PCs in the positive mode (*p* < 0.05). Differences in the coefficient of variation were significant for DGs, PCs, PEs, PE-Os, TGs, and others in the positive mode and PE-Os, PGs, PCs, and PEs in negative mode. The coefficients of variation for all of those were lower for the combined method than those of the reference. Basically, this indicates a higher specificity for the combined extraction method but not necessarily a higher efficiency. But, it was presumed that the homogenization with glass beads for the modified protocol would affect the extraction efficiency, by supporting the disruption of the epidermis of *C. elegans*. This effect seems to be more significant than the longer incubation time of 5 min than 1 h in the reference method. This is also the main difference that affects the CV positively. In addition, our results indicate that the MEB is not affecting the intensity of annotated features negatively.

**FIGURE 2:**
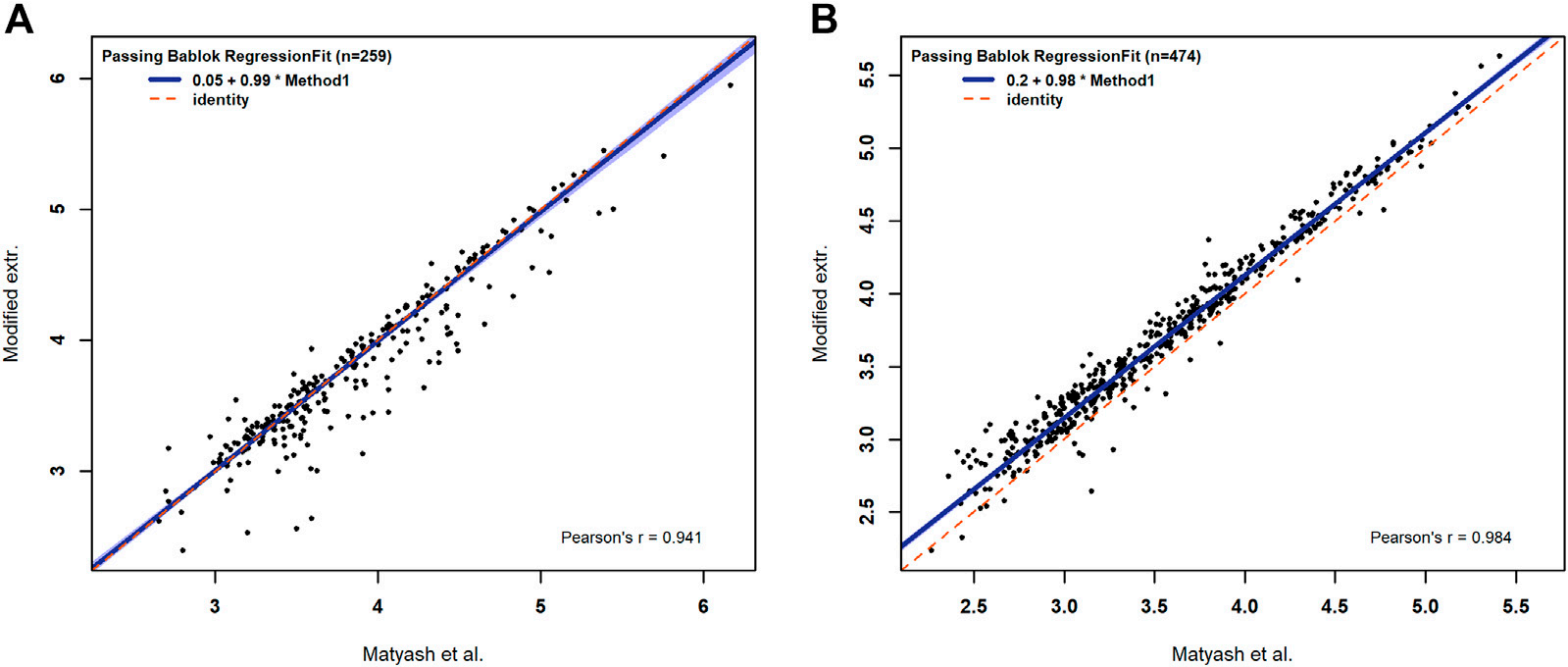
Passing–Bablok regression fits for the logarithmic intensities of identified lipids in negative **(A)** and in positive **(B)** modes. The dashed red line represents the half line with a slope of one and in blue is the the confidence band of the Passing and Bablok regression with an α of 0.05.

The comparison with the reference method for the number of annotations shows high agreement, as discussed before. Additionally, the intensities of all annotated lipids show a high level of positive agreement with *p* values <0.001 and *Pearson correlation coefficients* being 0.94 in the negative mode and 0.98 in the positive mode. The Passing and Bablok regression shows no significant difference between both methods (CI: 95%) regarding the slope and intercept in the negative mode, and no significant difference for the slope in the positive mode. In the positive mode, we obtained a significant shift to higher intensities for the combined method (Figure 2A,B). Nevertheless, the modifications made to the extraction method of Matyash et al. showed no negative effect on the lipid extraction efficiency and no effect on the lipid variety for the selected lipid classes. Therefore, a negative effect of the MEB on the extraction efficiency and variety can be excluded. Additionally, the modified method reduces the time spent on the extraction protocol and opens the possibility of simultaneous extraction of multiple samples. Our results clearly demonstrate that the incubation of 1 h in MeOH/MTBE, which is described in Matyash et al., is not necessary if a homogenization technique such as the cryo bead milling is used. The argon atmosphere in combination with BHT acts as a scavenger for oxygen radicals, and the low temperature prevents lipid oxidation during homogenization, which is necessary for the study of oxidized lipid species *in vivo* (Fujisawa, Kadoma et al., 2004). Furthermore, quartz beads are applied for the use in lipid extraction from *C. elegans,* which in this context has not been described before. Consequently, the modified extraction protocol is valid for the use of lipid extraction.

Even though, the modifications made to the extraction protocol including the use of the cryo unit and the additional steps to extract the aqueous phase are increasing the costs of the method and the amount of laboratory time are almost the same compared to Matyash et al. and 48 times less for the metal extraction. The modifications made are needed to keep the redox iron status stable and are shown to have no significant effect on the extraction efficiency compared to the reference method. Conclusively, the additional effort is necessary for the combined extraction of lipids and the metal species of interest.

### Metal Extraction

**FIGURE 3:**
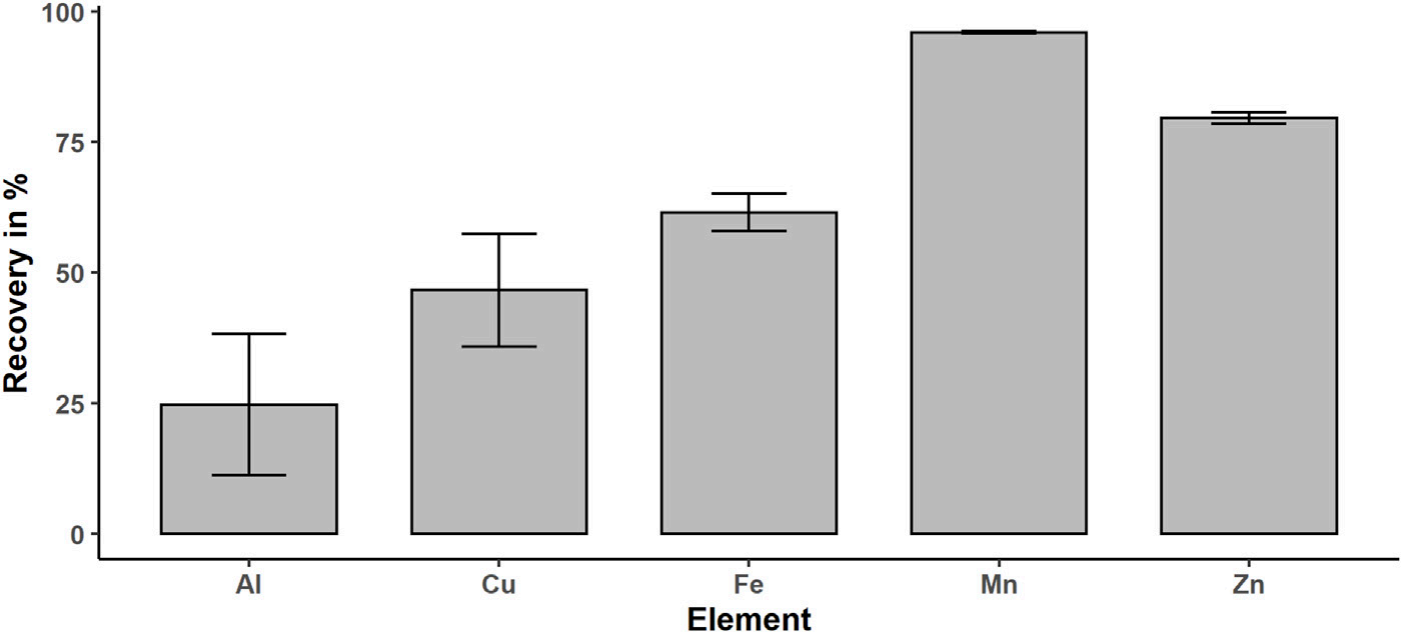
Obtained recoveries for extracted metals: Fe, Mn, Al, Cu, and Zn.

Based on the polar character of metal ions and EGTA complexes, the ions will sustain in the aqueous phase. Complexation with EGTA increases this effect and stabilizes the redox species Fe (II), which is susceptible to oxidation (Wilson and Carbonaro 2011). In addition, adsorption of Fe (III) on the surface of the quartz beads is minimized through complexing with EGTA by electrostatic repulsion. The determination of iron concentration in the organic phase with ICP-OES results in concentration level below the limit of quantification (LOQ) of 1 μg/L, whereas the aqueous extracts contain 18 ± 1.7 μg/L, which confirms that the aqueous phase is the metal enriched phase. The recovery of the extraction method was calculated by dividing the iron content of the extract by the total iron content, which is the sum of the extracts and digested pellet content, and multiplication with 100%. Therefore, we determined the metal concentration in the organic phase, the aqueous phase, and in the pellet–quartz beads mix after microwave digestion for 10 replicates. We achieved an iron recovery of 62 ± 8.2%, which is limited by remaining liquid in contact with the quartz beads and inclusions of metal in the remaining debris and precipitating proteins. If we take into account that we washed the pellet only one time after extraction to minimize the time at room temperature in the oxygen atmosphere, in respect of the susceptibility of Fe (II) to oxidation, this recovery is very acceptable. In addition, we determined the metal content in the aqueous phase and pellet for Al, Zn, Mn, and Cu in respect of their important function or toxicity in neurological disease progression (Figure 3) (Kozlowski, Luczkowski et al., 2012; Page, White et al., 2012; Guilarte 2013; Szewczyk 2013). Mn shows the highest recovery with 96% followed by Zn (80%), Fe (62%), Cu (47%), and Al (25%). The dependence on the recovery of the metal might be explained by the diversity of metal species. This extraction method is optimized for free ions, low molecular weight, and polar metal species. The membrane proteins such as cytochrome p450 oxidase (Šrejber, Navrátilová et al., 2018) will be partly removed by centrifugation, if the membrane disruption is incomplete. In addition, included or adsorbed metals in the debris and precipitating proteins due to the MeOH and MTBE in the extraction media will decrease the total recovery. However, the recovery of iron is acceptable, since we are interested in the free metal and low molecular metal species. For Mn, Zn, Al, and Cu, the recovery is very promising, and even though we optimized the protocol only for selected Fe and Mn species, these findings open new perspectives for future applications of this extraction protocol regarding speciation of metals other than Fe.

### Iron Redox State Speciation

**FIGURE 4:**
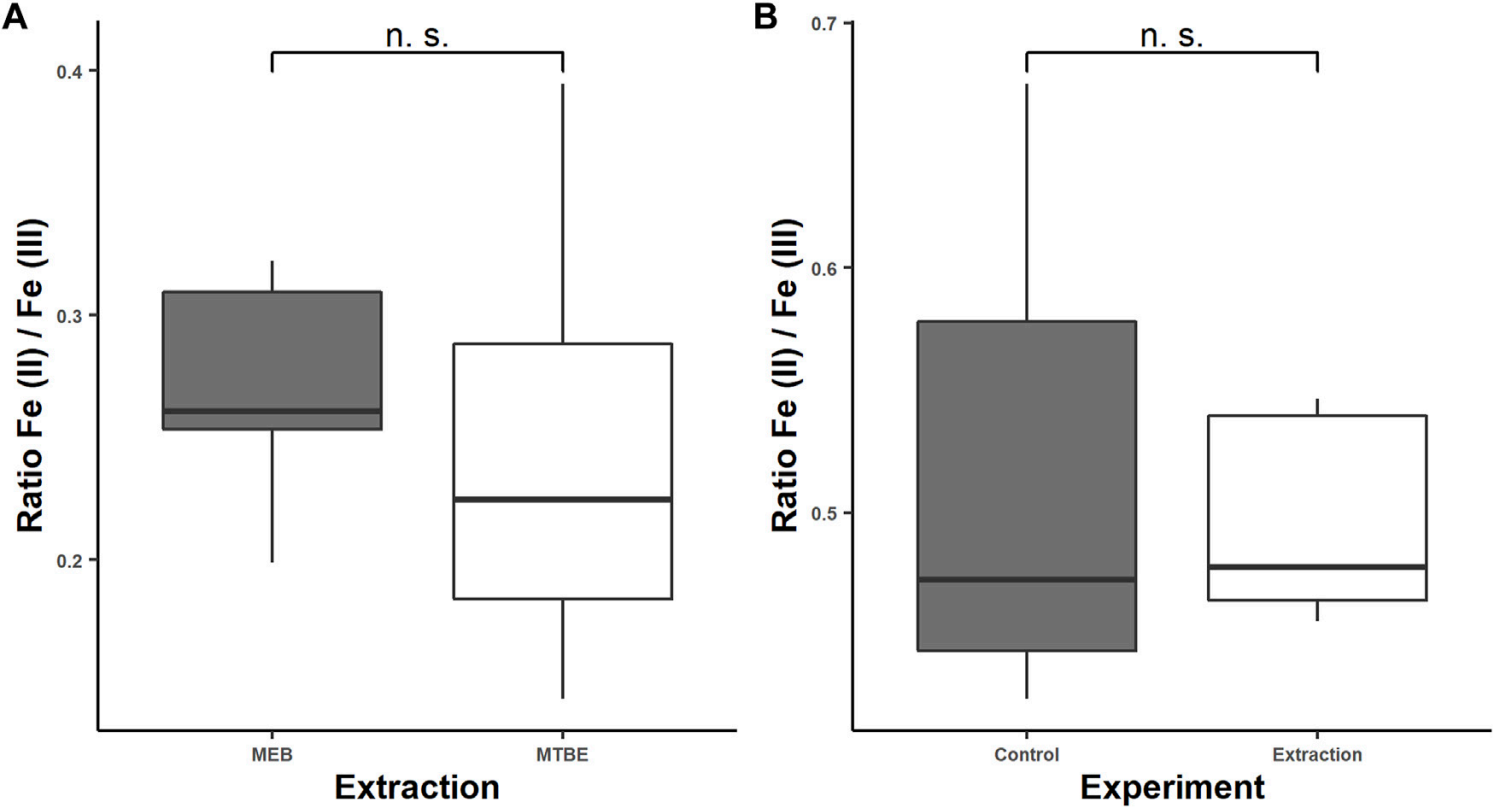
Fe (II)/Fe (III) ratios for the modified extraction protocol with and without traces of methyl-tert-butyl ether (MTBE) to study the effect of MTBE/MeOH on the iron redox state equilibrium **(A)** and Fe (II)/Fe (III) ratios for a 5-mg/L Fe (II) and Fe (III) standard solution in the MEB after following the extraction protocol compared to the standard solution **(B)**. The determination was carried out by CE–ICP-MS.

The sensitivity of Fe (II) to surrounding or solved oxygen is the major problem in iron redox state speciation. Under inappropriate storage conditions such as room temperature (RT), neutral pH, and air contact, Fe (II) oxidizes within minutes (Michalke, Willkommen et al., 2019). Therefore, it is required to exclude oxygen during sample preparation by the argon or nitrogen atmosphere and keep the pH low. By flushing all solvents with Ar and overlaying the caps with Ar or N_2_, the excluding of oxygen during extraction was achieved. In order to extract the sample close to the physiological pH (intracellular: 7–7.4) to keep metal species close to the native environment, EGTA and BHT were added, which have a stabilizing effect on the Fe (II) species as demonstrated previously (Wilson and Carbonaro 2011). The complexation of Fe (II) and Fe (III) with EGTA has two effects. First, it inhibits the electron transfer by sterical stabilization, and second it prevents Fe (III) to form hydroxyl complexes, which accelerate the oxidation of Fe (II) (Morgan and Lahav 2007). In contrast to ethylenediaminetetraacetic acid (EDTA), which acts like a catalyst for the *Haber–Weiss* reaction, EGTA showed no catalytic activity (Sutton 1985). Additionally, EGTA prevents the iron species from adsorbing at the surface of the quartz beads and capillary wall by electrostatical repulsion of the negatively charged complex Fe-EGTA^-/2-^. The adsorption to the capillary wall occurs through the high specific surface area of about 8 m^2^ l^−1^ for a capillary of 100 cm length and 50 µm ID, which we identified as a major problem in iron redox state speciation. Additionally, the dynamic coating with CTAB and reversed current prevent adsorption of Fe (II) and Fe (III) to free silanol groups on the capillary surface. The BHT scavenges free radicals, such as the hydroxyl radical, which would alter the iron redox state equilibrium by *Haber–Weiss* reaction as mentioned before (Black 2002). The extraction media components such as EGTA, NaCl, and MOPS as well as the snap-freezing do not alter the iron redox status as demonstrated before (Diederich and Michalke 2011; Wilson and Carbonaro 2011). However, organic solvents during the extraction and remaining organic solvents in the aqueous phase might cause an alteration of the iron redox status during extraction or have an effect on the iron determination with CE–ICP-MS. Therefore, we studied the effect of MeOH and MTBE remaining in the aqueous phase on the Fe (II)/Fe (III) ratio compared with an extract containing only the MEB. Accordingly, the extraction was performed five times using a mixed culture of *C. elegans* as a model organism, following our modified extraction without adding organic solvents and straight according to the protocol. We determined the area under the curve (AUC) for Fe (II) and Fe (III), which we identified by standard addition. The observed average ratios were 0.27 ± 0.06 for the MTBE free extraction and 0.25 ± 0.11 for the extract with MTBE (Figure 4A).

**FIGURE 5:**
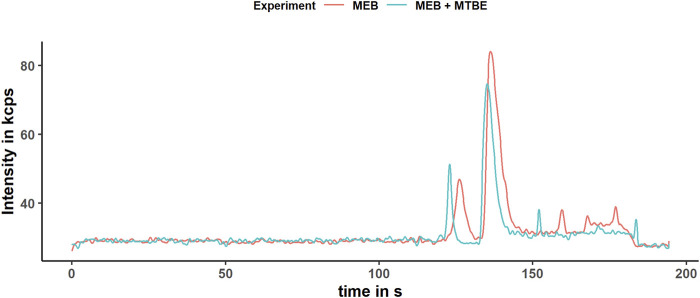
Electropherogram of the extracts obtained according to the modified protocol with (blue) and without (red) organic solvent.The electropherogram was obtained after smoothing the data by applying the *Savitzky–Golay* filter. The determined isotope was Fe-56 in the DRC-ammonia mode.

Lipid precipitation was observed for the extraction without organic solvents, which might lead to accelerated capillary alteration and therefore should be avoided (Dawod, Arvin et al., 2017). The modified homogenization technique using the glass beads and high frequency of shaking might alter the iron redox state. Therefore, we proved the stability of iron redox state during extraction by determining the iron redox state equilibrium of five 5 mg/L standard solutions Fe (II)/Fe (III) in the MEB after following the extraction protocol and comparing it to five 5 mg/L standard solutions before extraction. The samples and control were stored under the same condition and time under argon, frozen in liquid nitrogen for transport, and were measured directly after the extraction, to prevent time-related oxidation of Fe (II). The Fe (II)/Fe (III) ratio for the standard solutions after extraction was 0.49 ± 0.02 and for the control 0.52 ± 0.04 (Figure 4B). Consequently, we proved the stability of the iron species during the extraction and showed that MTBE and MeOH do not affect the extraction of the iron species or interfere the determination with CE–ICP-MS. Additionally, a slight change in the peak shape was noticed, which was caused by the remaining MTBE and MeOH in the aqueous phase toward more narrow peaks (Figure 5). This positive effect improved the resolution of performed measurements. However, a decrease in the AUC was noticed, which is explained by the narrowing peak width, while the peak height was constant. The lower integrated signal might explain the higher variation of the Fe (II)/Fe (III) ratio, since this is proportional to the amount of data points generated for each peak. The limit of generated data points was set by the dwell time of 50 ms and the number of measured isotopes (Fe-56 and Fe-57). A reduction in the dwell time might result in less variation, but it will also decrease the intensity and LODs. For our purpose, we already optimized this parameter regarding variation and intensity, but this will differ depending on the metal concentration in the sample.

Traces of remaining oxidative species, such as H_2_O_2_, formed by UV-C radiation (Piskarev 2018), might alter the iron species, and we noticed problems to keep the iron species stable, especially in the standard solutions. This, we solved by using low temperature, freshly prepared buffer storage in the dark, BHT as an antioxidant, EGTA as a complexing agent, Ar atmosphere, and short extraction times. The final composition of 6 mM NaCl, 5 mM MOPS, and 1 mM EGTA in the MEB was based on the previous work (Wilson and Carbonaro 2011) and slightly modified to match the ionic strength of the electrolyte used in the CE determination. In summary, the extraction media are stabilizing the labile iron redox status and optimizing the performance in the following determination process. Interestingly, the biological matrix seemed to stabilize the iron species. The reason for that observation is likely the cellular ROS defense mechanisms, such as superoxide dismutase in combination with catalase and glutathione peroxidase and glutathion (GSH) as an electron donor (Hanukoglu 2006). Additionally, GSH forms Fe (II)-GSH complexes at a physiological pH, which are discussed to be responsible for the stabilization of free iron (II) in the labile iron pool (Hider and Kong 2011). This gives some evidences why the iron redox state in biological samples is more stable than the iron standard in the MEB. In summary, the main differences to the extraction protocol of Matyash et al. (Matyash, Liebisch et al., 2008) are the extraction solvents, the liquid nitrogen freezing, the use of a cryo unit, and glass bead homogenizer as well as Ar-flushed solvents. These modifications are essential for the stability of the redox iron status during the extraction and the performance of the following determination. Additionally, the extraction time is reduced by a factor of 48 compared to the existing protocol (Diederich and Michalke 2011), since up to 24 samples can be homogenized simultaneously in 5 min compared to only one sample using a glass Dounce homogenizer under the Ar atmosphere in 10 min.

## Conclusion

We have successfully established a modification of the lipid extraction protocol previously described from Matyash et al. (Matyash, Liebisch et al., 2008) for the new application in iron redox state speciation for the model organism *C. elegans*. The results showed similar extraction efficiencies compared to the reference method for the important lipid classes. We reduced the laboratory time spent on the extraction and opened up the opportunity to extract multiple samples simultaneously for the extraction of the labile iron redox equilibrium. Using this combined protocol, we enabled the extraction of lipid species as well as free iron and other metals simultaneously. Additionally, lipid and protein precipitation in the aqueous phase as well as associated alteration of the capillary, caused by protein adsorption on the silica surface, is prevented. We reduced the alteration of the iron species during extraction, with the combined effect of the MEB buffer, argon atmosphere, BHT, and liquid nitrogen cooling. Consequently, the determination of free metal species and the ratio between iron (II) and iron (III) together with lipid species from the same sample was successfully established.

## Data Availability

The raw data supporting the conclusions of this article will be made available by the authors, without undue reservation.
